# Method for detecting acetylated PD-L1 in cell lysates by immunoprecipitation and western blot analysis

**DOI:** 10.1371/journal.pone.0268887

**Published:** 2022-07-18

**Authors:** Frances Middleton-Davis, Ashley Davis, Kim Middleton

**Affiliations:** R&D Department, Cytoskeleton Inc., Denver, Colorado, United States of America; Centre de Recherche en Biologie cellulaire de Montpellier, FRANCE

## Abstract

Lysine acetylation is an important regulatory post-translational modification (PTM) that occurs sub-stoichiometrically, often representing less than 1% of the target protein. This makes studying endogenous protein acetylation extremely challenging. Recent reports suggest that several post-translational modifications (PTMs), including lysine acetylation, play a major role in the regulation the programmed cell death-ligand 1 (PD-L1), a clinically important protein target. An enrichment step is necessary to enable identification of the acetylated species by either antibody or mass spectrometry-based detection methods. This report describes a robust lab protocol for the enrichment and detection of endogenous acetylated PD-L1 protein. A recently developed acetyl lysine affinity matrix was utilized to enrich >90% of acetylated PD-L1 species, from a variety of cell lines, spanning a fourteen-fold range of target protein levels. Western blot analysis, using a highly sensitive PD-L1 antibody and optimized transfer times, was used to determine that the endogenous level of acetylated PD-L1 is in the range of 0.02–0.07% of total PD-L1. As validation, we demonstrate that acetylation levels increase to 0.11–0.17% of total PD-L1 after a 4h treatment with the histone deacetylase (HDAC) inhibitor trichostatin A (TSA). The method described here is simple to perform in any lab equipped with tissue culture and western blot equipment.

## Introduction

Immune checkpoint therapy targeting PD-L1 protein has shown remarkable results in the treatment of a variety of cancers. However, the response within patient cohorts varies considerably, making it clear that a thorough mechanistic understanding of PD-L1 regulation is lagging the clinical success of this target. It is anticipated that such advances will lead to more efficacious clinical use and novel therapies [1].

Lysine acetylation is a key regulator of protein function and is involved in cell processes such as transcription, metabolism, and signal transduction [2]. Acetylation of PD-L1 was first reported by Horita et al. [3] where the acetylated species was detected in response to epidermal growth factor stimulation of A431 cells; suggesting a dynamic regulation of PD-L1 by acetylation in this system. Studies by Gao et al. suggest that de-acetylation of PD-L1 at lysine 263 is essential for its nuclear translocation and for the modulation of gene expression affecting tumor aggressiveness and responsiveness to immune checkpoint therapy [4]. The authors suggest that blocking PD-L1 de-acetylation, and hence nuclear translocation, with HDAC inhibitors may have therapeutic potential [4]. Conversely, it has been reported, in breast cancer models, that acetylation of PD-L1 at lysine 270 can stabilize the protein and accelerate breast tumor growth [5]. In this case, the authors suggest that a strategy of blocking PD-L1 acetylation could be a valid therapeutic approach [5]. HDAC inhibitors have also been shown to alter the tumor microenvironment and enhance tumor immunogenicity and have been proposed as a combinatorial therapy with immune checkpoint inhibitors [6]. While progress has been made in establishing that acetylation is likely to be an important regulator of PD-L1, it is not yet clear how therapeutic approaches, that alter global acetylation, will affect PD-L1 function in specific cancers, tumor environments or patient cohorts and warrants further investigation [7]. There is also a growing interest in elucidating if and how other PTMs such as ubiquitination, glycosylation, and phosphorylation crosstalk with acetylation to regulate PD-L1 activity [7–9]. The ability to quantitate endogenous levels of acetylated PD-L1under various experimental conditions is an important tool to help with these efforts.

Here we present a modified protocol of Horita et al. [3]. The method allows quantitation of PD-L1 acetylation and is applicable to a wide range of cell lines. It is simple to perform in any laboratory equipped with tissue culture and western blot equipment. The method involves cell lysate preparation, IP enrichment of acetylated PD-L1 using an acetyl-lysine affinity matrix and western blot detection of both total and acetylated PD-L1 using a PD-L1 antibody. The lysis buffer used is described in Horita et al. [3] and has been optimized for disruption of protein:protein interactions, solubilization of semi-denatured proteins, efficient lysis of all cellular compartments and the stabilization and efficient immunoprecipitation (IP) of acetylated species. We have improved the method of Horita et al in several ways; (1) The highly sensitive anti-PD-L1 antibody used in this protocol was found to be critical for optimal detection of the acetyl-PD-L1 signal. (2) The acetyl-lysine affinity beads contain antibodies that have a higher affinity for acetylated PD-L1 and have been made using an improved crosslinking technique resulting in very clean IP results, making the method user-friendly for labs that do not routinely perform IPs. (3) IP incubation times, wash steps and western blot transfer times have been optimized for enhanced detection of low-level acetylated PD-L1.

This technique compliments the alternative IP approach utilizing a PD-L1 antibody as the IP reagent and an anti-acetyl lysine antibody as the detection reagent. However, because the protocol described here enables the detection of both total and acetylated PD-L1 on the same blot, this method has the advantage of allowing an estimate of the percent of PD-L1 that is acetylated, an important parameter for mechanistic interpretation.

Unlike quantitative mass spectrometry, this method does not have the potential to identify and quantitate specific endogenous acetylation sites. The specialized equipment and high level of expertise that quantitative mass spectrometry of sub-stoichiometric species demands is out of reach for most laboratories and is technically demanding even for specialist labs; currently the only reported quantitative mass spectrometry of a PD-L1 PTM is the abundant modification of N-glycosylation [10]. The development of acetylated PD-L1 specific antibodies would enable analysis of specific modifications, in this regard there are ongoing attempts to develop and validate these reagents which would be useful in determining relative changes in acetylation [4, 5]. We have determined that >90% of available acetylated PD-L1 species are captured using this protocol and therefore classify the method as semi-quantitative. It also remains possible that there are some buried acetyl-PD-L1 species that cannot be captured by this technique. In this regard, acetylation of the relatively unstructured cytoplasmic tail of PD-L1, which contains five potential acetylation sites, is a major focus of current research [4, 5] and these are predicted to be highly available to this IP technique. The method described here is simple, semi-quantitative and enables labs working on widely different aspects of PD-L1 biology to quickly assess if acetylation plays a role in their system of study.

## Materials

The protocol described in this article is published on protocols.io, https://dx.doi.org/10.17504/protocols.io.bxgcpjsw.

### Expected results

**Fig 1:**
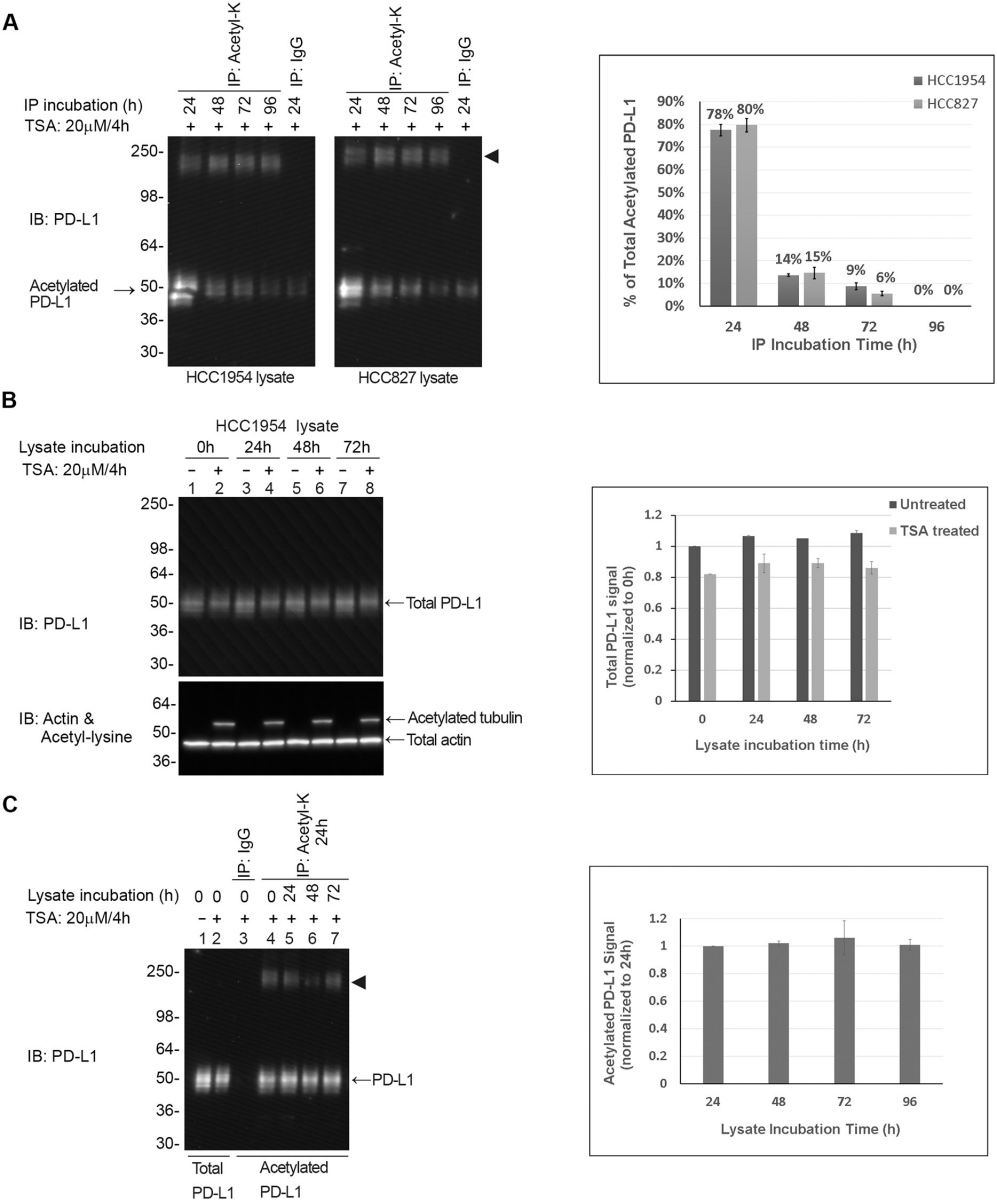
Assay efficiency in capturing >90% of acetylated PD-L1 in cell lysates. (A) Cell lines were grown to 50% confluency and treated for 4h with 20 μM trichostatin A. Lysates (1 mg) were incubated with acetyl lysine enrichment beads (Acetyl-K) for 24h (Lane 1), 48h (Lane 2), 72h (Lane 3) and 96h (Lane 4). After each 24h incubation period beads were pelleted, and fresh Acetyl-K enrichment beads were added to lysates. Acetylated proteins were eluted from Acetyl-K beads and were resolved by SDS-PAGE. Acetylated PD-L1 was identified using an anti-PD-L1 antibody. The arrowhead represents a crosslinked protein G: antibody complex that leaches off the Acetyl-K affinity beads. Lane 5 shows background PD-L1 signal from mouse IgG control beads (IgG). Acetylated PD-L1 signals (minus IgG background signal) were quantitated by densitometry using imageJ software and the percent of acetylated PD-L1 in each IP fraction was calculated (graph 1A). (B) HCCT1954 cells were grown to 50% confluency and either treated with 20 μM TSA for 4h (Lanes 2,4,6,8) or untreated (Lanes 1,3,5,7). Lysates were incubated for 24h, 48h or 72h at 4ºC after which 1 ug of fresh lysate (0h, Lanes 1 & 2)) or incubated lysates (Lanes 3–8) were resolved by SDS-PAGE total PD-L1 was identified using an anti-PD-L1 antibody (Lanes 1–8, Total PD-L1). Total PD-L1 signals were quantitated by densitometry using imageJ software, levels of total PD-L1 were expressed relative to the PD-L1 protein levels of fresh lysate (0h, Lanes 1&2) levels are shown in graph Fig 1B. The PD-L1 blot was re-probed with anti-actin antibody (Total actin) to confirm gel loading consistency and with anti-acetyl lysine antibody to confirm enhanced acetylation in TSA treated lysates as indicated by acetylated tubulin bands (Acetylated tubulin). (C) HCC1954 cells were grown to 50% confluency and treated for 4h with 20 μM TSA. Lysates were harvested and either immediately snap frozen in liquid nitrogen and stored at -80ºC (0h, Lane 4) or incubated at 4ºC for 24h (Lane 5), 48h (Lane 6) or 72h (Lane 7) after which lysates (1mg) were incubated for 24h with acetyl lysine affinity beads (IP: Acetyl-K). The enriched protein fractions were resolved by SDS-PAGE acetylated PD-L1 was identified using an anti-PD-L1 antibody. The arrowhead represents a crosslinked protein G: antibody complex that leaches off the acetyl lysine affinity beads. Acetylated PD-L1 signals were quantitated by densitometry using imageJ software and levels of acetylated PD-L1 were expressed relative to the acetylated PD-L1 protein levels of fresh lysate (0h, Lane 4) levels are shown in graph 1C. Shown are representative westerns from N≥3 independent experiments.

The protocol described can be used to estimate the percent of PD-L1 that is acetylated in any given cell lysate. The enrichment step requires a 48h incubation period with 70–80% of acetylated PD-L1captured over 24h and >90% after 48h (Fig 1A). Importantly, the assay buffer composition maintains the stability of total and acetylated PD-L1 over the course of the experimental timeline (Fig 1B & 1C).

**Fig 2:**
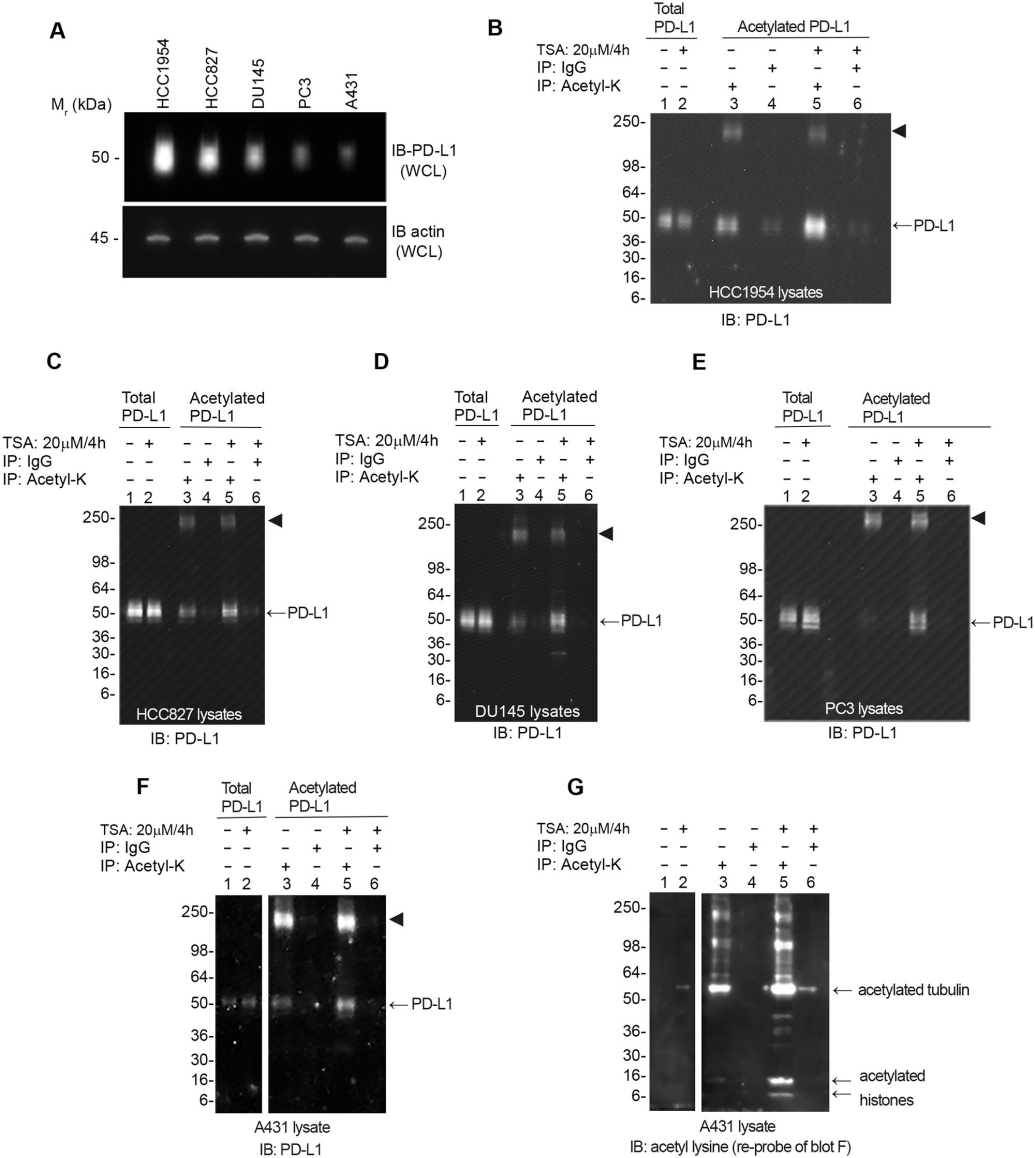
Detection of enhanced acetylation of PD-L1 with HDAC inhibitor treatment. (A). Cell lines were grown to 50% confluency. Cell lysates (1 μg per sample) were run on SDS-PAGE and analyzed by western blot for total PD-L1. Actin was used as a loading control. (B-F) Cells were grown to 50% confluency and either treated with 20 μM TSA for 4 hours (Lanes 2, 5 & 6) or untreated (Lanes 1, 3 & 4). Lysates were incubated with either acetyl-lysine enrichment beads (Acetyl-K; Lanes 3 & 5) or mouse IgG control beads (IgG; Lanes 4 & 6). The enriched protein fractions were resolved by SDS-PAGE acetylated PD-L1 was identified using an anti-PD-L1 antibody. Total lysate input (Total PD-L1) was run for untreated (Lane 1), and TSA treated (Lane 2) lysates and total PD-L1 was identified using an anti-PD-L1 antibody. Acetylated (Lanes 3–6) and total (Lanes 1–2) PD-L1 signals were quantitated by densitometry using imageJ software and the percent of acetylated PD-L1 was calculated. Percentages are reported in Table 1. The arrowhead represents a crosslinked protein G: antibody complex that leaches off the Acetyl-K affinity beads. The following amounts of IP lysate & input lysates (Lanes 1 & 2) were used (B) HCC1954; IP 0.5 mg, input 0.2 μg. (C) HCC827; IP 0.5 mg, input 0.5 ug. (D) DU145; IP 0.5 mg, input 0.5 ug (E) PC3; IP 1.0 mg, input 1.0 μg. (F) A431; IP 1.0 mg, input 0.4 μg. (G) The western blots from (F) were re-probed with anti-acetyl lysine antibody. Shown are representative westerns from N≥3 independent experiments.

The assay was used to examine cell lines spanning a 14-fold range of PD-L1 protein levels (Table 1, Fig 2A). Baseline levels of acetylated PD-L1, for cell lines grown to 50% confluency in manufacturer recommended growth media, were detected, and quantitated in all cell lines studied (Table 1, Fig 2B–2F), supporting the utility of the assay to determine changes in acetylation levels under varying experimental conditions. To further validate the assay PD-L1 acetylation levels were quantitated after a four-hour treatment with the histone deacetylase (HDAC) inhibitor trichostatin A (TSA) (Fig 2B–2F). In all cell lines studied the percent of PD-L1 acetylation increased over baseline by 2.2–6.6 fold depending upon the cell line used.

**Table 1:** Profile of PD-L1 acetylation in a variety of cell lines.

Cell Line	Tissue Source	Relative levels of total cellular PD-L1 protein*	TSA stimulation of total PD-L1	Baseline PD-L1 acetylation (% of total)	Acetyl PD-L1 in TSA treated extracts (% of total)	Fold increase in PD-L1 acetylation after TSA treatment
HCC1954	Breast duct	1.00	0.77 ±0.07	0.043±0.002	0.17±0.03	4.0x ±0.45
HCC827	Lung	0.6 ±0.02**	0.83 ±0.08	0.050±0.010	0.11±0.01	2.2x ±0.30
DU145	Prostate	0.29 ±0.02	1.03 ±0.02	0.030±0.010	0.12±0.04	4.0x ±0.30
PC3	Prostate	0.13 ±0.02	1.37 ±0.03	0.022±0.008	0.14±0.04	6.6x ±0.6
A431	Skin	0.07 ±0.02	1.27 ±0.23	0.070±0.010	0.14±0.01	2.1x ±0.15

*Relative to untreated HCC1954 lysate

**standard error of the mean

Re-probing the western blot with a pan anti-acetyl lysine antibody (Fig 2G) allows visualization of the total protein acetylation profile in any given lysate, a property that is useful when examining PD-L1 acetylation in the presence of HDAC inhibitors or other treatments affecting global acetylation.

## Supporting information

S1 File Step by step protocol, also available on protocols.io. (PDF)
