# Supplementary material for: Method for detecting acetylated PD-L1 in cell lysates by immunoprecipitation and western blot analysis
Source: PLoS One. 2022 Jul 18;17(7):e0268887. doi: 10.1371/journal.pone.0268887 (PMC9292098; doi:10.1371/journal.pone.0268887)
Supplement: S1 File — (PDF) [file pone.0268887.s001.pdf]

S2: Supporting Information for Figure 1, Figure 2 and Table 1

Support for Fig 1A: Assay Efficiency in Capturing >90% of Acetylated PD-L1 in Cell Lysates

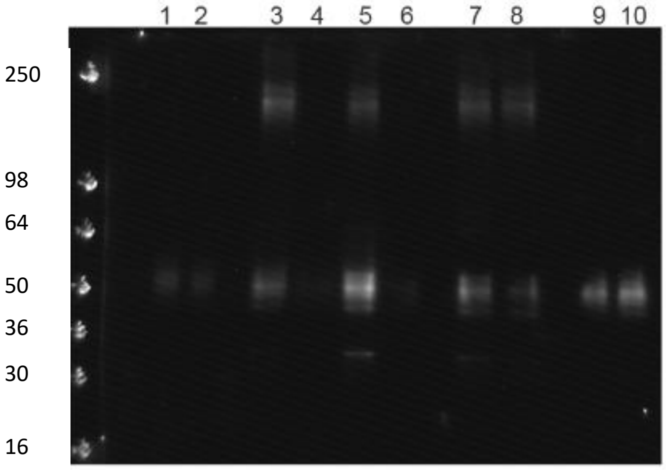

DU145: acetyl PD-L1 IP Time course

Lane	Sample
1	untreated (UT) input: 0.2 ug
2	TSA treated input: 0.2 ug
3	UT-AcetylK-24h
4	UT-IgG-24h
5	TSA-AcetylK-24h
6	TSA-IgG-24h
7	TSA-AcetylK-48h
8	TSA-AcetylK-72h
9	TSA-whole cell lysate (WCL) supernatant 72h
10	TSA WCL-input

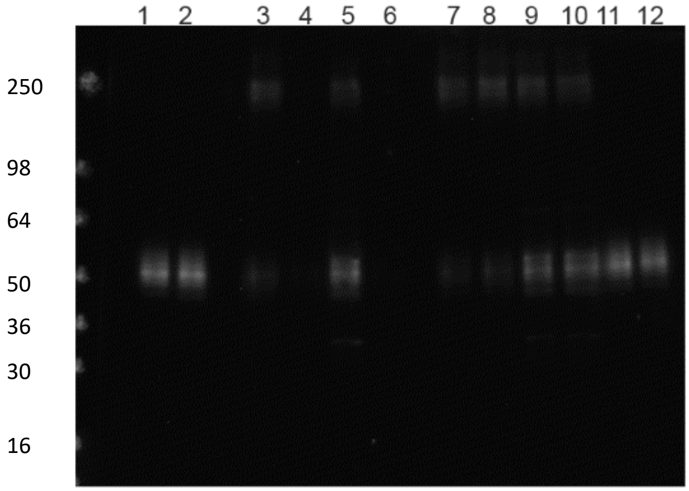

DU145: acetyl PD-L1 IP Time course

Lane	Sample
1	untreated (UT) input: 0.5 ug
2	TSA treated input: 0.5 ug
3	UT-AcetylK-24h
4	UT-IgG-24h
5	TSA-AcetylK-24h
6	TSA-IgG-24h
7	UT-AcetylK-48h
8	UT-IgG followed by AcetylK-48h
9	TSA-AcetylK-48h
10	TSA:IgG followed by AceylK-48h
11	UT-0.5ug 48h supernatant
12	TSA-0.5 ug 48h supernatant

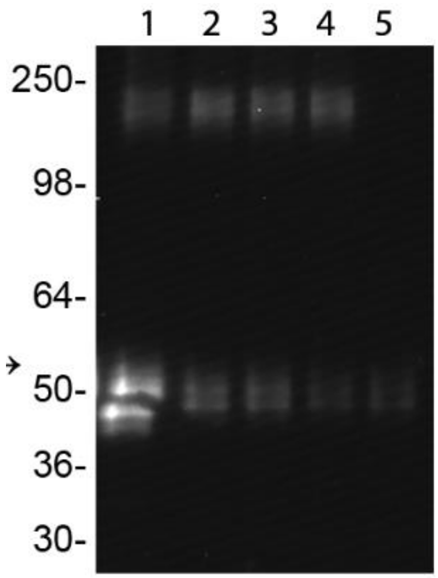

HCC1954: acetyl PD-L1 IP Time course

Lane	Sample
1	TSA-AcetylK-24h
2	TSA-AcetylK-48h
3	TSA-AcetylK-72h
4	TSA-AcetylK-96h
5	TSA-IgG-24h

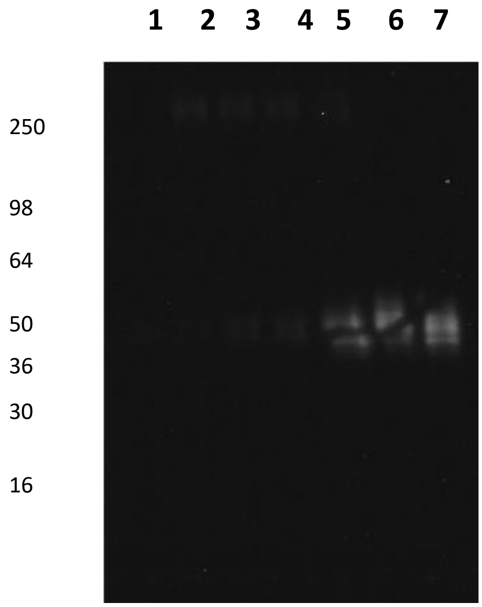

HCC1954: acetyl PD-L1 IP Time course

Lane	Sample
1	TSA-IgG-72h
2	TSA-IgG-48h
3	TSA-IgG-24h
4	TSA-AcetylK-96h
5	TSA-AcetylK-72h
6	TSA-AcetylK-48h
7	TSA-AcetylK-24h

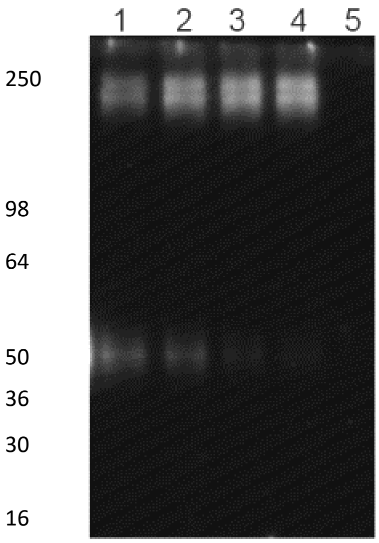

DU145: acetyl PD-L1 IP Time course

Lane	Sample
1	TSA-AcetylK-24h
2	TSA-AcetylK-48h
3	TSA-AcetylK-72h
4	TSA-AcetylK-96h
5	TSA-IgG-24h

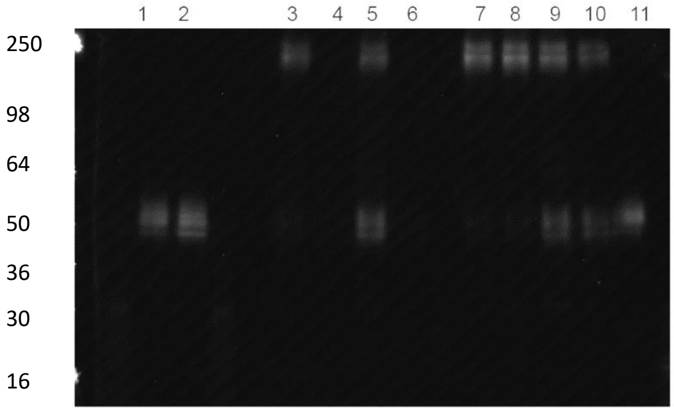

PC3 : acetyl PD-L1 IP Time course

Lane	Sample
1	untreated (UT) input: 0.5 ug
2	TSA treated input: 0.5 ug
3	UT-AcetylK-24h
4	UT-IgG-24h
5	TSA-AcetylK-24h
6	TSA-IgG-24h
7	UT-AcetylK-48h
8	UT-IgG-48h
9	TSA-AcetylK-48h
10	TSA-IgG followed by AcetylK-48h
11	UT input-48h

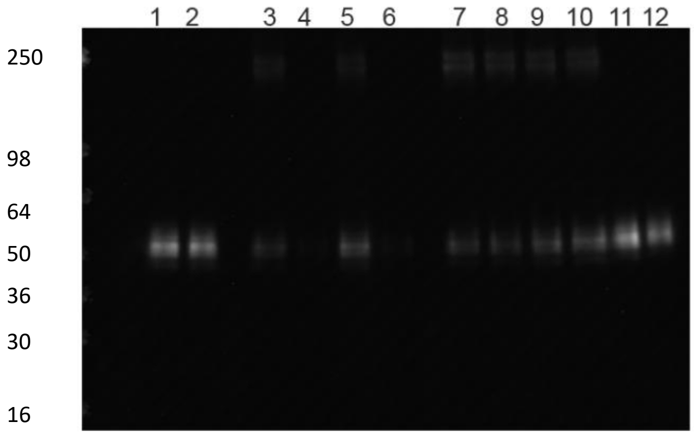

HCC827 : acetyl PD-L1 IP Time course

Lane	Sample
1	untreated (UT) input: 0.5 ug
2	TSA treated input: 0.5 ug
3	UT-AcetylK-24h
4	UT-IgG-24h
5	TSA-AcetylK-24h
6	TSA-IgG-24h
7	UT-AcetylK-48h
8	UT-IgG followed by AcetylK-48h
9	TSA-AcetylK-48h
10	TSA-IgG followed by AcetylK-48h
11	UT-WCL-0.5ug -48h
12	TSA-WCL-0.5ug-48h

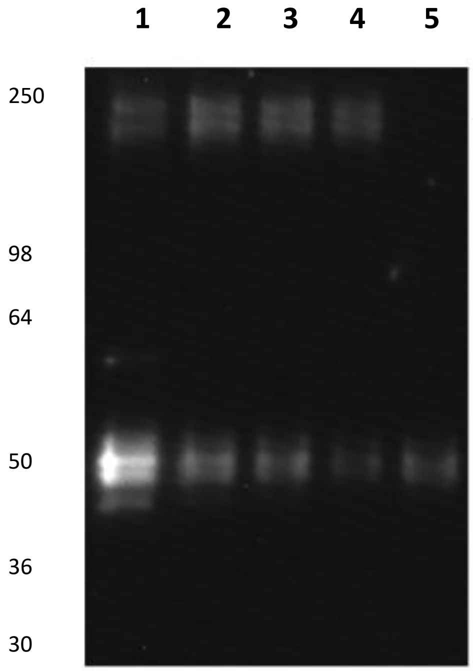

HCC827: acetyl PD-L1 IP Time course

Lane	Sample
6	TSA-AcetylK-24h
7	TSA-AcetylK-48h
8	TSA-AcetylK-72h
9	TSA-AcetylK-96h
10	TSA-IgG-24h

Support for Fig 1B: Stability of PD-L1 over a 72h timecourse

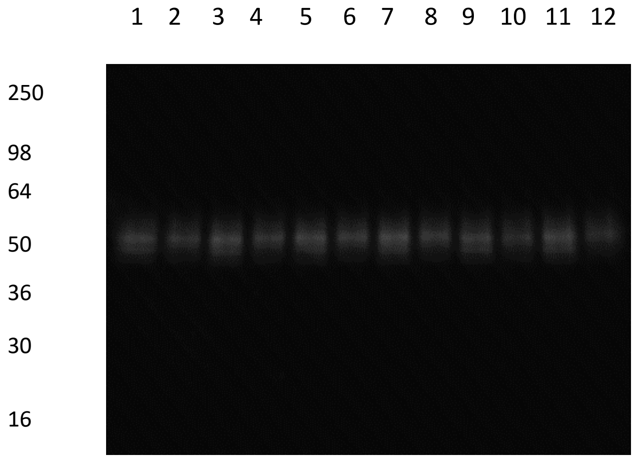

HCC1954: PD-L1 stability over time

Lane	Sample
1	UT-0h
2	TSA-0h
3	UT-24h
4	TSA-24h
5	UT-48h
6	TSA-48h
7	UT-72h
8	TSA-72h
9	UT-0h
10	TSA-0h
11	UT-72h
12	TSA-72h

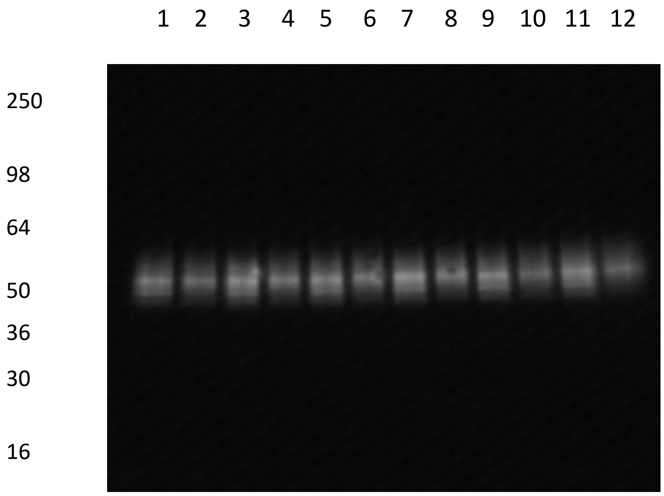

HCC1954: PD-L1 stability over time

Lane	Sample
13	UT-0h
14	TSA-0h
15	UT-24h
16	TSA-24h
17	UT-48h
18	TSA-48h
19	UT-72h
20	TSA-72h
21	UT-0h
22	TSA-0h
23	UT-72h
24	TSA-72h

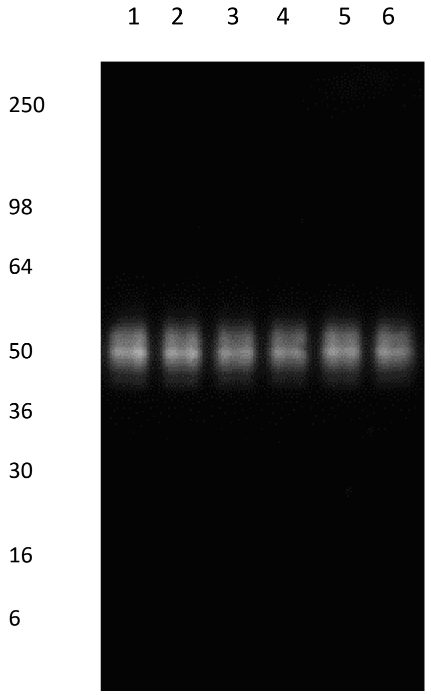

DU145: PD-L1 stability over time

Lane	Sample
1	UT-0h
2	TSA-0h
3	UT-24h
4	TSA-24h
5	UT-48h
6	TSA-48h

<u>Support for Fig 1C: Stability of acetylated PD-L1 over a 72h timecourse</u>

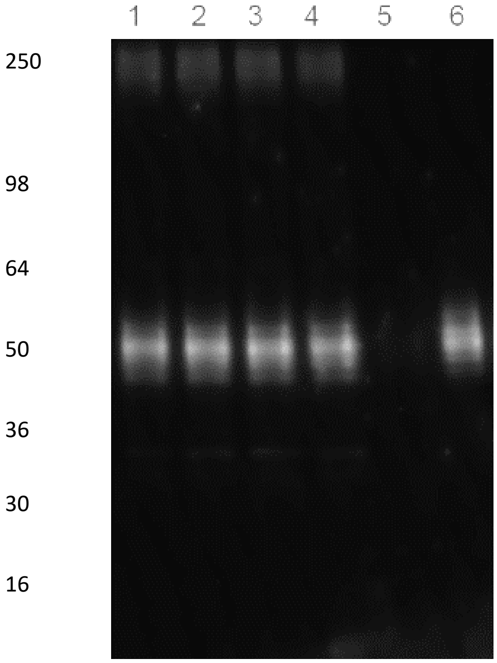

HCC827: Lysate time course for acetylated PD-L1 stability

Lane	Sample
1	TSA-AcetylK-72h lysate/24h IP
2	TSA-AcetylK-48h lysate/24h IP
3	TSA-AcetylK-24h lysate/24h IP
4	TSA-AcetylK-0h lysate/24h IP
5	TSA-IgG-0h/24h IP
6	TSA input: 0.5ug

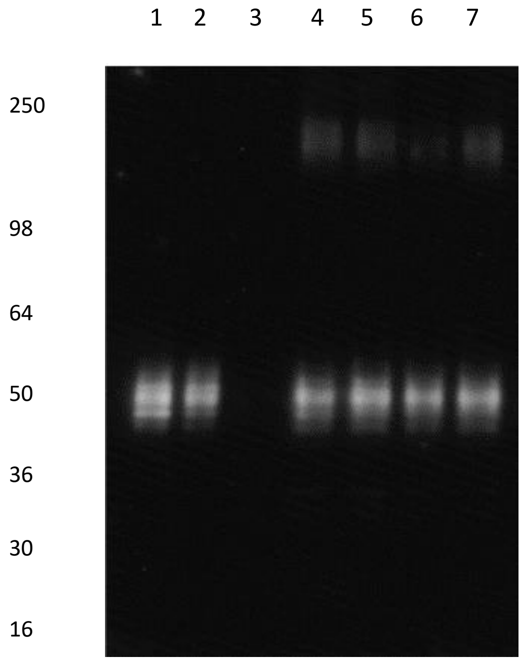

HCC1954: Lysate time course for acetylated PD-L1 stability

Lane	Sample
1	UT input 1ug
2	TSA input 1ug
3	TSA-IgG-0h lysate/24h IP
4	TSA-AcetylK-0h lysate/24h IP
5	TSA-AcetylK-24h lysate/24h IP
6	TSA-AcetylK-48h lysate/24h IP
7	TSA-AcetylK-72h lysate/24h IP

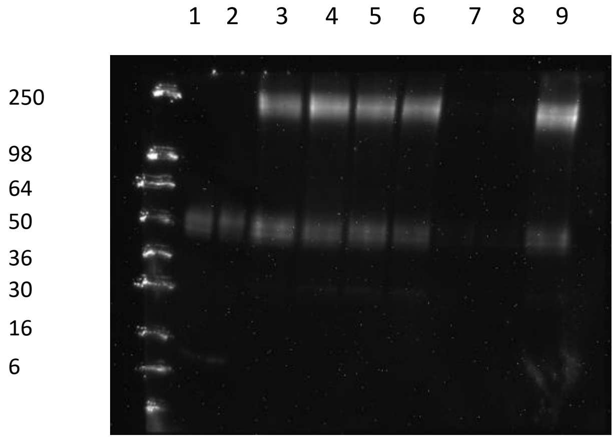

HCC1954: Lysate time course for acetylated PD-L1 stability

Lane	Sample
1	UT input-0.5ug
2	TSA input-0.5ug
3	TSA-AcetylK-0h lysate/24h IP
4	TSA-AcetylK-24h lysate/24h IP
5	TSA-AcetylK-48h lysate/24h IP
6	TSA-AcetylK-72h lysate/24h IP
7	TSA-IgG-0h lysate/24h IP
8	TSA-IgG-72h lysate/24h IP
9	TSA-AcetylK-0h lysate/24h IP

Support for Fig 2 and Table 1

Support for Fig 2A & Table 1: Relative levels of PD-L1 in a variety of cell lines

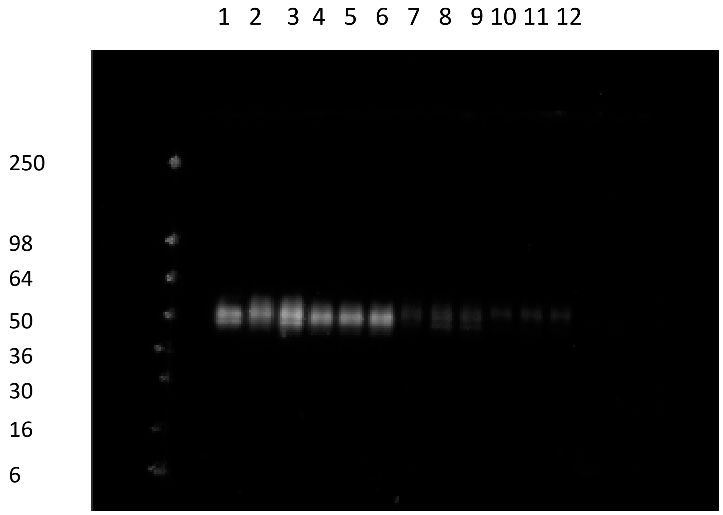

NOTE: Lanes 3,6,9 &12 are SCA treated lysates and are not part of this study.

PD-L1 levels in various Cell Lines

Lane	Sample
1	HCC1954-UT-1ug
2	HCC1954-TSA-1ug
3	HCC1954-SCA-1ug
4	HCC827-UT-1ug
5	HCC827-TSA-1ug
6	HCC827-SCA-1ug
7	PC3-UT-1ug
8	PC3-TSA-1ug
9	PC3-SCA-1ug
10	A431-UT-1ug
11	A431-TSA-1ug
12	A431-SCA-1ug

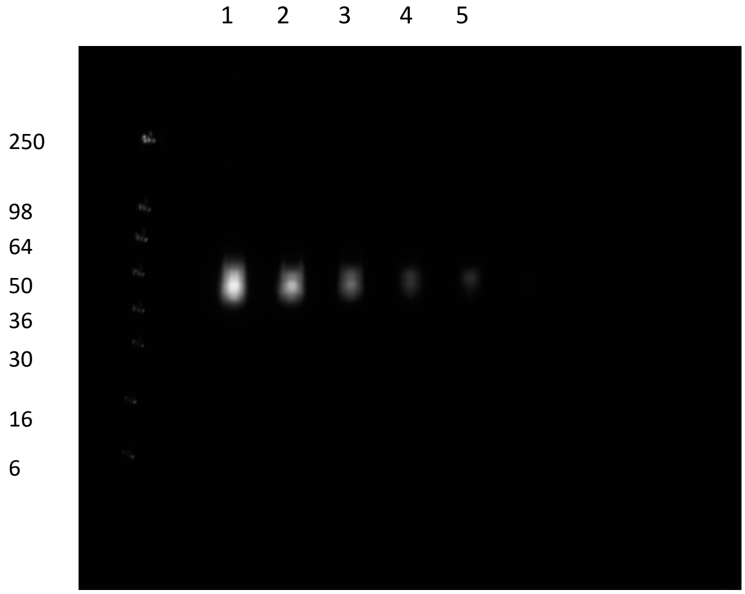

PD-L1 levels in various Cell Lines

Lane	Sample
1	HCC1954-UT-1ug
2	HCC827-1ug
3	DU145-1ug
4	PC3-1ug
5	A431-1ug

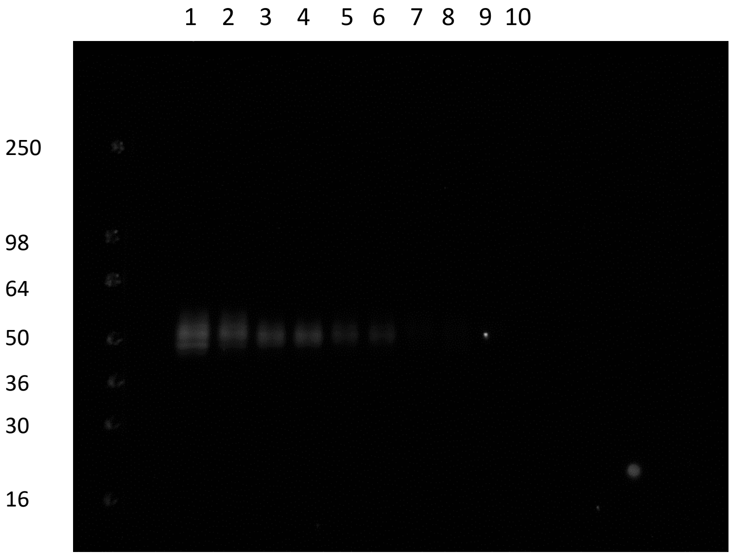

PD-L1 levels in various Cell Lines

Lane	Sample
1	HCC1954-UT-1ug
2	HCC1954-TSA-1ug
3	HCC827-UT-1ug
4	HCC827-TSA-1ug
5	DU145-UT-1ug
6	DU145-TSA-1ug
7	PC3-UT-1ug
8	PC3-TSA-1ug
9	A431-UT-1ug
10	A431-TSA-1ug

Support for Fig 2B-F & Table 1: Detection of changes in PD-L1 Acetylation in response to TSA (HDAC inhibitor)

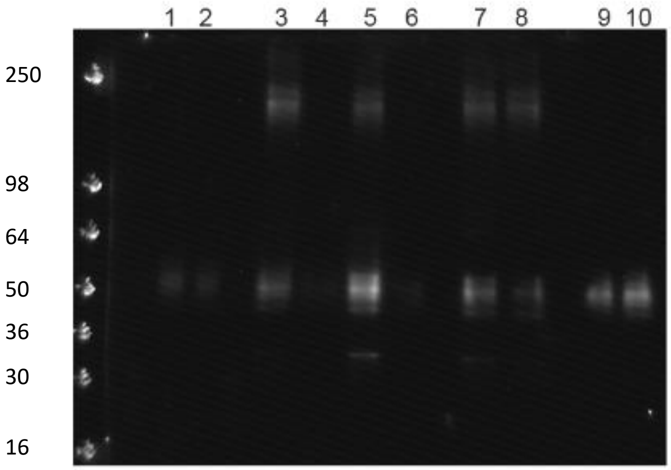

DU145: PD-L1 Acetylation Changes in response to TSA

Lane	Sample
1	untreated (UT) input: 0.2 ug
2	TSA treated input: 0.2 ug
3	UT-AcetylK-24h
4	UT-IgG-24h
5	TSA-AcetylK-24h
6	TSA-IgG-24h
7	TSA-AcetylK-48h
8	TSA-AcetylK-72h
9	TSA-whole cell lysate (WCL) supernatant 72h
10	TSA WCL-input

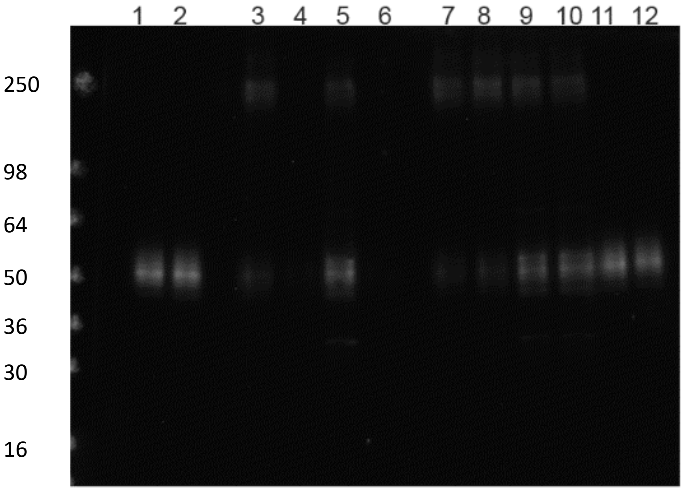

DU145: PD-L1 Acetylation Changes in response to TSA

Lane	Sample
1	untreated (UT) input: 0.5 ug
2	TSA treated input: 0.5 ug
3	UT-AcetylK-24h
4	UT-IgG-24h
5	TSA-AcetylK-24h
6	TSA-IgG-24h
7	UT-AcetylK-48h
8	UT-IgG followed by AcetylK-48h
9	TSA-AcetylK-48h
10	TSA:IgG followed by AceylK-48h
11	UT-0.5ug 48h supernatant
12	TSA-0.5 ug 48h supernatant

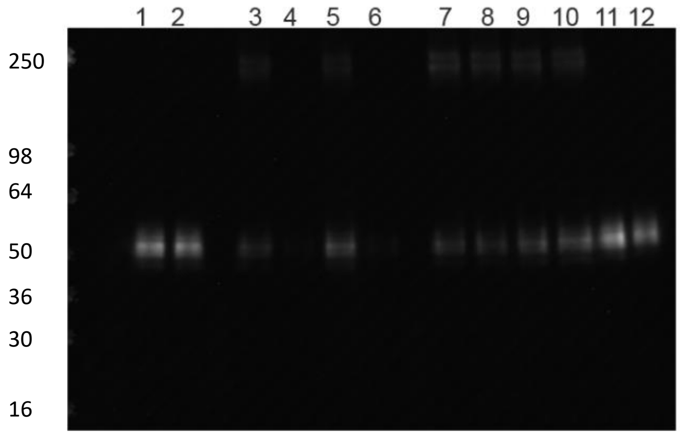

HCC827: PD-L1 Acetylation Changes in response to TSA

Lane	Sample
1	untreated (UT) input: 0.5 ug
2	TSA treated input: 0.5 ug
3	UT-AcetylK-24h
4	UT-IgG-24h
5	TSA-AcetylK-24h
6	TSA-IgG-24h
7	UT-AcetylK-48h
8	UT-IgG followed by AcetylK-48h
9	TSA-AcetylK-48h
10	TSA-IgG followed by AcetylK-48h
11	UT-WCL-0.5ug -48h
12	TSA-WCL-0.5ug-48h

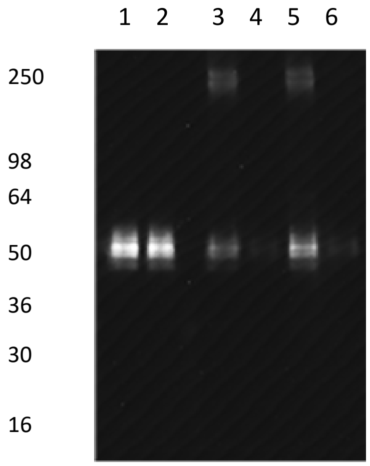

HCC827: PD-L1 Acetylation Changes in response to TSA

Lane	Sample
1	UT input-1 ug
2	TSA input-1ug
3	UT-AcetylK-24h
4	UT-IgG-24h
5	TSA-AcetylK-24h
6	TSA-IgG-24h

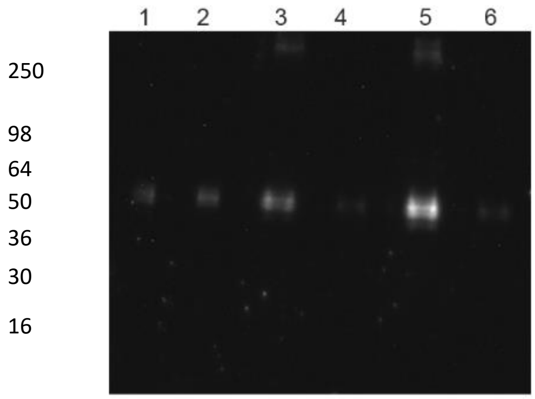

HCC827: PD-L1 Acetylation Changes in response to TSA

Lane	Sample
1	UT input-0.2 ug
2	TSA input-0.2ug
3	UT-AcetylK-24h
4	UT-IgG-24h
5	TSA-AcetylK-24h
6	TSA-IgG-24h

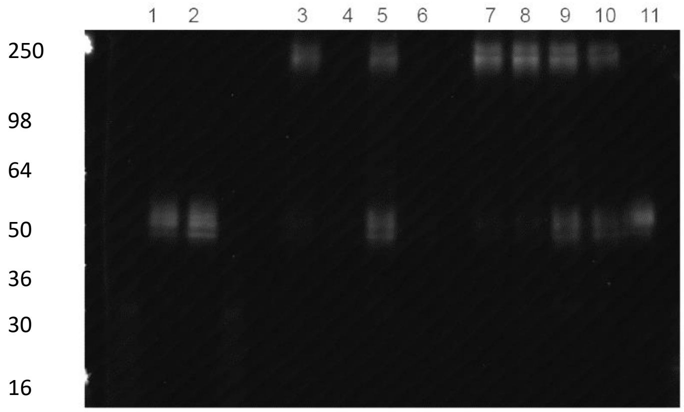

PC3: PD-L1 Acetylation Changes in response to TSA

Lane	Sample
1	untreated (UT) input: 0.5 ug
2	TSA treated input: 0.5 ug
3	UT-AcetylK-24h
4	UT-IgG-24h
5	TSA-AcetylK-24h
6	TSA-IgG-24h
7	UT-AcetylK-48h
8	UT-IgG-48h
9	TSA-AcetylK-48h
10	TSA-IgG followed by AcetylK-48h
11	UT input-48h

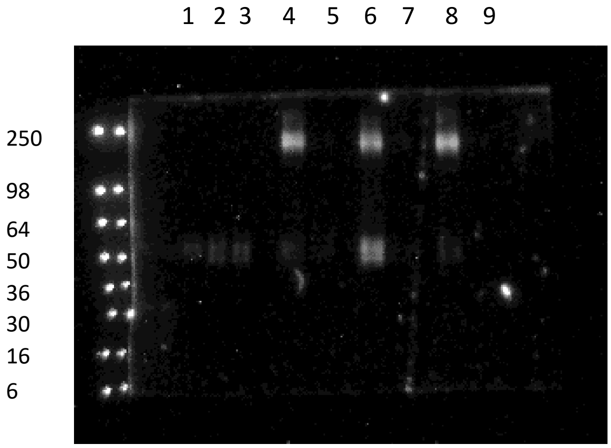

PC3: PD-L1 Acetylation Changes in response to TSA

Lane	Sample
1	untreated (UT) input: 0.2ug
2	TSA treated input: 0.2 ug
3	SCA treated input: 0.2ug
4	UT-AcetylK-24h
5	UT-IgG-24h
6	TSA-AcetylK-24h
7	TSA-IgG-24h
8	SCA-AcetylK-48h
9	SCA-IgG-48h

NOTE: Lanes 3,8 & 9 are SCA treated lysates and are not part of this study.

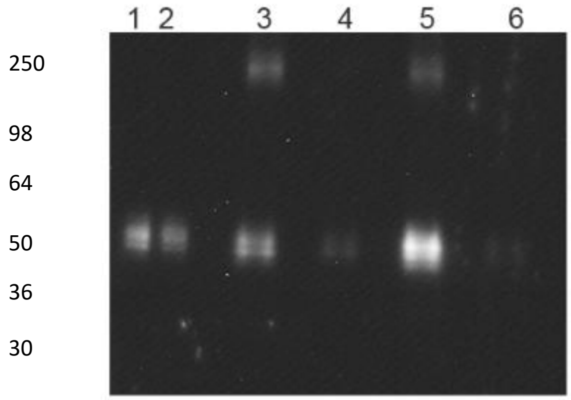

HCC1954: PD-L1 Acetylation Changes in response to TSA

Lane	Sample
1	UT input-0.2 ug
2	TSA input-0.2ug
3	UT-AcetylK-24h
4	UT-IgG-24h
5	TSA-AcetylK-24h
6	TSA-IgG-24h

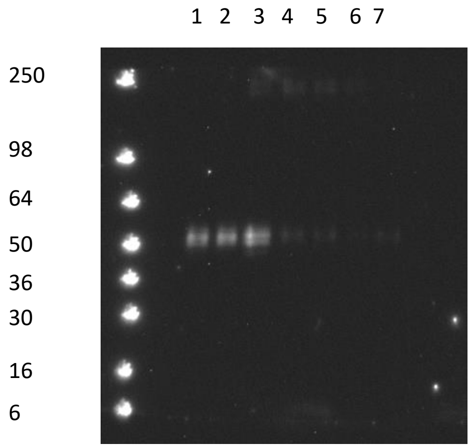

HCC1954: PD-L1 Acetylation Changes in response to TSA

Lane	Sample
1	TSA input-0.2 ug
2	UT input-0.2ug
3	TSA-AcetylK-24h
4	UT-AcetylK-24h
5	TSA-Acetyl-48h
6	UT-Acetyl-48h
7	TSA-IgG-24h

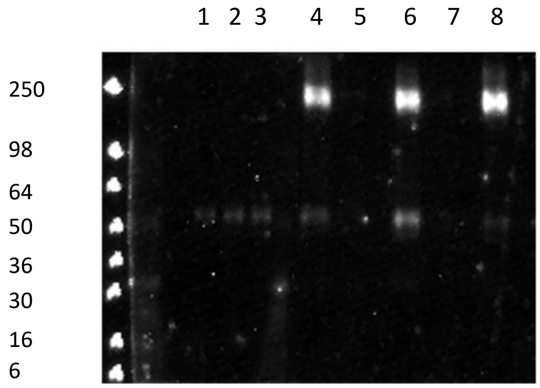

A431: PD-L1 Acetylation Changes in response to TSA

Lane	Sample
1	UT input-2 ug
2	TSA input-2ug
3	TSA input-2ug
4	UT-AcetylK-24h
5	UT-IgG-24h
6	TSA-AcetylK-24h
7	TSA-IgG-24h
8	UT-AcetylK-24h

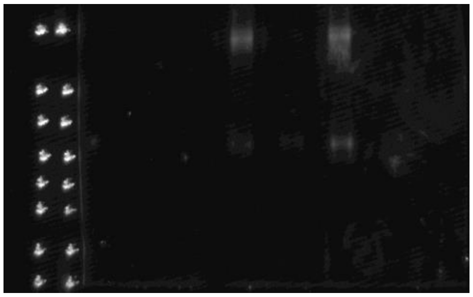

A431: PD-L1 Acetylation Changes in response to TSA

Lane	Sample
1	UT-AcetylK-24h
2	UT-IgG-24h
3	TSA-AcetylK-24h
4	TSA-IgG-24h
